# DIGITAL TECHNOLOGY USE AND SOCIAL CAPITAL: THE MODERATING EFFECT OF SOCIAL CLASS

**DOI:** 10.1093/geroni/igac059.2185

**Published:** 2022-12-20

**Authors:** Bomi Choi, Hayoung Park, Susanna Joo, Yoon-Myung Kim, Hyoun K Kim

**Affiliations:** Yonsei University, Seoul, Seoul-t'ukpyolsi, Republic of Korea; Yonsei University, Seoul, Seoul-t'ukpyolsi, Republic of Korea; Yonsei University, Seoul, Seoul-t'ukpyolsi, Republic of Korea; Yonsei University, Seoul, Seoul-t'ukpyolsi, Republic of Korea; Yonsei University, Seoul, Seoul-t'ukpyolsi, Republic of Korea

## Abstract

This study aims to elucidate the heterogeneous associations between digital technology use and social capital by social class. The sample comprises 315 Korean older people who are 65 years old or older and participated in an online survey. Digital technology use was measured by the frequency of independent use in four areas of digital technology: primary, cultural, economic, and public areas. Social capital was measured with ten items asking the perceived support availability from both online and offline relationships. Social class was measured with education, household income, and subjective social position to reflect both objective and subjective aspects of social class. Using SPSS 25 PROCESS Macro 3.5, linear regression with moderation analyses was performed. A simple slope and the region of significance were tested for a significant interaction. Results showed that subjective social position significantly moderated the relationship between digital technology use and social capital. The positive association between digital technology use and social capital was strengthened when the level of subjective social position was higher. Education and household income did not moderate the relationship between digital technology use and social capital. The results of this study indicate that the effects of digital technology use vary depending on perceived aspects of social class. This study also demonstrates that people with higher social classes enjoy more benefits from digitalization, supporting digital inequality among the older population.

